# Exome Sequencing Identifies a Missense Variant in *EFEMP1* Co-Segregating in a Family with Autosomal Dominant Primary Open-Angle Glaucoma

**DOI:** 10.1371/journal.pone.0132529

**Published:** 2015-07-10

**Authors:** Donna S. Mackay, Thomas M. Bennett, Alan Shiels

**Affiliations:** Department of Ophthalmology and Visual Sciences, Washington University School of Medicine, St. Louis, Missouri, United States of America; Radboud University Nijmegen Medical Centre, NETHERLANDS

## Abstract

Primary open-angle glaucoma (POAG) is a clinically important and genetically heterogeneous cause of progressive vision loss as a result of retinal ganglion cell death. Here we have utilized trio-based, whole-exome sequencing to identify the genetic defect underlying an autosomal dominant form of adult-onset POAG segregating in an African-American family. Exome sequencing identified a novel missense variant (c.418C>T, p.Arg140Trp) in exon-5 of the gene coding for epidermal growth factor (EGF) containing fibulin-like extracellular matrix protein 1 (*EFEMP1*) that co-segregated with disease in the family. Linkage and haplotype analyses with microsatellite markers indicated that the disease interval overlapped a known POAG locus (GLC1H) on chromosome 2p. The p.Arg140Trp substitution was predicted *in silico* to have damaging effects on protein function and transient expression studies in cultured cells revealed that the Trp140-mutant protein exhibited increased intracellular accumulation compared with wild-type EFEMP1. *In situ* hybridization of the mouse eye with oligonucleotide probes detected the highest levels of EFEMP1 transcripts in the ciliary body, cornea, inner nuclear layer of the retina, and the optic nerve head. The recent finding that a common variant near *EFEMP1* was associated with optic nerve-head morphology supports the possibility that the *EFEMP1* variant identified in this POAG family may be pathogenic.

## Introduction

Glaucoma is a clinically heterogeneous group of optic neuropathies that present as progressive loss of visual field, with or without elevated intraocular pressure, characteristic excavation ('cupping') of the optic nerve head as a result of retinal ganglion cell death [1]. Worldwide, glaucoma constitutes a prevalent cause (~3.54%) of irreversible blindness afflicting over 64 million adults aged 40–80 years [2]. Primary open-angle glaucoma (POAG), in which the irido-corneal angle and anterior eye structures appear normal under gonioscopy examination, is the most common form diagnosed in all populations studied and is especially prevalent (~4.2%) in those with African ancestry. Genetic approaches reveal that POAG may be inherited either as a common, complex trait with adult onset or, less frequently, as a classical Mendelian or monogenic disease that tends to have an early onset [3] (OMIM, www.omim.org).

Genetic linkage studies of multiplex families, mostly of European ancestry, have identified at least 21 loci (GLC) for Mendelian forms of POAG [4–8]. Four of these loci (GLC3A-D) have been linked with autosomal recessive primary congenital or infantile glaucoma (PCG), 15 loci (GLC1A-H, GLC1J, GLC1K-N, GLC1P-Q) with juvenile-onset (10–35 years) and/or adult-onset (>35 years) forms of autosomal dominant POAG, and two loci with adult-onset, complex POAG (GLC1I, GLC1O). So far, linkage-based approaches have resulted in the discovery of eight causative genes for monogenic POAG namely, *MYOC* (GLC1A), *OPTN* (GLC1E), *ASB10* (GLC1F), *WDR36* (GLC1G), *NTF4* (GLC1O), *TBK1* (GLC1P), *CYP1B1* (GLC3A), and *LTBP2* (GLC3D). However, the identity of causative genes at the remaining loci remains enigmatic.

Beyond linkage studies, numerous (>120) case-control association studies of candidate-gene or genome-wide common genetic variants have sought to identify susceptibility genes for adult-onset, complex POAG [7]. Currently, single nucleotide polymorphisms (SNPs) and/or copy number variations (CNVs) in at least 65 possible susceptibility genes or loci have been identified for complex POAG predominantly in populations of Caucasian and Asian ancestries. Such genetic heterogeneity is consistent with multiple risk variants, each with small pathogenic effects, contributing to POAG etiology. It has been estimated that variants in at least five of the genes identified through linkage studies of Mendelian POAG (*MYOC*, *OPTN*, *WDR36*, *CYP1B1*, *ASB10*) may account for up to 10% of the heritability of complex POAG cases, suggesting that discovery of additional genes for monogenic forms of POAG may enhance understanding of the genetic architecture of complex POAG. Moreover it appears that genetic risk variants for the disproportionally high prevalence of POAG in anthropologically-older populations of African ancestry may differ from those in Caucasians [9,10]. Here we have conducted trio-based, whole-exome, massively-parallel sequencing in order to identify the genetic mutation underlying an autosomal dominant form of POAG segregating in an African-American family.

## Materials and Methods

### Ethics statement

Ethical approval for this study was obtained from the Washington University Human Research Protection Office and written informed consent was provided by all participants prior to enrollment in accordance with the tenets of the Declaration of Helsinki, and Health Insurance Portability and Accountability Act (HIPAA) regulations. Procurement of animal tissue was approved by the Washington University Animal Studies Committee and conformed to the guidelines published by the Institute for Laboratory Animal Research.

### Family participants

**Fig 1:**
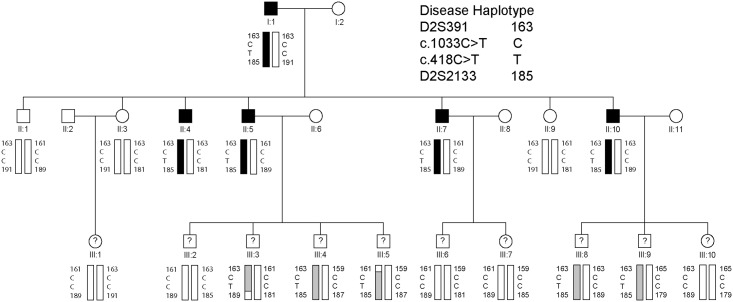
Three-generation African-American family segregating autosomal dominant primary open-angle glaucoma. Pedigree and haplotype analysis showing segregation of microsatellite (D2S) markers and single nucleotide variants in *EFEMP1* (c.418C>T, c.1033C>T) listed in descending physical order from the short-arm telomere of chromosome 2 (2p-tel). Squares denote males and circles denote females. In the first and second generations, filled symbols denote individuals with confirmed affected status and filled bars denote the disease haplotype. In the third generation, question marks denote individuals of unconfirmed disease status and shaded bars indicate those with the disease haplotype. The trio of individuals I:1, II:4, and II:9 was selected for whole exome sequencing.

An African-American family segregating autosomal dominant POAG was ascertained through ophthalmic records in the Department of Ophthalmology and Visual Sciences at Washington University School of Medicine. Blood samples were obtained from available family members including five affected males, one unaffected male, two unaffected females, and ten individuals in the third generation of unknown disease status (Fig 1). Leukocyte genomic DNA was purified using the Gentra Puregene Blood kit (Qiagen, Valencia, CA), and quantified by absorbance at 260 nm (NanoDrop 2000, Wilmington, DE).

### Exome sequencing

Whole exome capture was achieved using the SureSelect Human All Exon V5 (50.4 Mb) Kit, according to manufacturer’s instructions (Agilent Technologies, Santa Clara, CA). Briefly, genomic DNA (3 μg) was fragmented (150–200 bp) by acoustic shearing, ligated to adapter primers, and PCR-amplified. Following denaturation (95°C, 5 min), amplified DNA-fragment libraries (~500 ng) were hybridized in-solution under high stringency (65°C, 24 hr) with biotinylated RNA capture probes (~120 bp). Resulting DNA/RNA hybrids were recovered by streptavidin-coated magnetic bead separation (Dynal, Invitrogen, Calsbad, CA). Captured DNA was eluted (NaOH) and then subject to flow-cell massively-parallel sequencing on a HiSeq2000 System (Illumina, San Diego, CA) using the Illumina Multiplexing Sample Preparation Oligo-nucleotide Kit, and the HiSeq 2000 Paired-End Cluster Generation Kit according to the manufacturer’s instructions. Briefly, hybrid-capture libraries were amplified to add indexing (identifying) tags and sequencing primers then subjected to paired-end (2 x 101 bp read length), multiplex sequencing-by-synthesis using fluorescent, cyclic reversible (3’-blocked) terminators. A pool of three exome samples (representing a family trio) was sequenced in a single lane of the sequencer’s flow-cell.

### Exome variant analysis

Raw sequence data was aligned to the human reference genome (build hg19) by NovoalignMPI (www.novocraft.com), and sequence variants called using the Sequence Alignment/Map format (SAMtools) and Picard programs (http://samtools.sourceforge.net/) and further annotated using SeattleSeq (http://snp.gs.washington.edu/SeattleSeqAnnotation131/). Target coverage and read-depth were reviewed by the Integrated Genomics Viewer (IGV, http://www.broadinstitute.org/igv/) (S1 Table). Called variants were reviewed using the SNP & Variation Suite 8 software (Golden Helix, Bozeman, MT) and the Ingenuity Variant Analysis (IVA) website (http://ingenuity.com). Potential disease causing variants were evaluated by a four-step process. First, variants were selected based on co-segregation with disease in the family trio (Fig 1) and all other variants were excluded. Second, we excluded those variants co-segregating with disease in the trio that were also present in public genome variant databases including; dbSNP (http://www.ncbi.nlm.nih.gov/snp/), 1000 genomes (http://www.1000genomes.org/), and the Exome Variant Server (EVS, http://evs.gs.washington.edu/EVS/). Third, the remaining variants were validated by Sanger sequencing in the trio and analyzed *in silico* for effect on protein function using the SIFT (http://sift.jcvi.org) and PolyPhen-2 (http://genetics.bwh.harvard.edu/pph2/) mutation-prediction programs. Finally, validated variants from the trio were tested for co-segregation with disease in the rest of the family by Sanger sequencing (S2 and S3 Tables).

### Sanger Sequencing

Genomic DNA (2.5 ng/μl, 10 μl reactions), was amplified (35 cycles) in a GeneAmp 9700 thermal cycler using Top Taq mastermix kit (Qiagen) and 20 pmol of gene-specific primers (S4 Table) [11]. Resulting PCR amplicons were enzyme-purified with ExoSAP-IT (USB Corporation, Cleveland, OH). Purified amplicons were direct cycle-sequenced in both directions with BigDye Terminator Ready Reaction Mix (v3.1) (Applied Biosystems/Life Technologies, Grand Island, NY) containing M13 forward or reverse sequencing primers, then ethanol precipitated and detected by capillary electrophoresis on a 3130xl Genetic Analyzer running Sequence Analysis (v.6.0) software (Applied Biosystems), and Chromas (v2.23) software (Technelysium, Tewantin, Queensland, Australia).

### Microsatellite genotyping and linkage analysis

Microsatellite markers from the National Center for Biotechnology Information (NCBI) combined Généthon, Marshfield, and deCODE genetic linkage maps (www.ncbi.nlm.nih.gov/genome/guide/human/) were genotyped with size markers (GeneScan 600 LIZ dye Size Standard v2.0) by capillary electrophoresis on a 3130xl Genetic Analyzer running fragment-analysis software (GeneMapper Software 5), according to the maufacturer’s instructions(Applied Biosystems). Pedigree and haploptype data were managed using Cyrillic (v. 2.1) software (FamilyGenetix Ltd., Reading, UK), and two-point LOD scores (Z) calculated using the MLINK sub-program from the LINKAGE (5.1) package of programs (http://linkage.rockefeller.edu/soft/) (S5 Table). Marker allele frequencies were assumed to be equal. A frequency of 0.01% and a penetrance of 100% were assumed for the disease allele.

### Cell culture and plasmid transfection

HEK-293T cells (ATCC CRL-3216 purchased May 9, 2014 from American Type Culture Collection, Manassas, VA) were cultured (37°C, 5% CO_2_) in Dulbecco’s modified Eagle’s medium (DMEM) containing 4.5 g/L glucose, 2 mM L-glutamine, and sodium pyruvate (Fisher Scientific, Waltham, MA), and supplemented with 10% fetal bovine serum (Gibco Life Technologies) and 1% penicillin/streptomycin (Fisher Scientific). Human EFEMP1 reference and mutant (c.418C>T) cDNA sequences (GenBank accession no. NM_001039348.2) were custom synthesized and directionally sub-cloned into the pReceiver-M13 vector carrying a C-terminal fusion FLAG-tag (GeneCopoeia, Rockville, MD) and the resulting plasmids verified by Sanger sequencing. Plasmid DNA (10 μg) was transfected into HEK-293T cell monolayers in 60 mm dishes (70–90% confluence) using Lipofectamine 2000 reagent (Invitrogen/Life Technologies, Carlsbad, CA) in OptiMEM 1 reduced serum media (Invitrogen) for 4 hr and then cultured for a further 24–48 hr in fresh reduced serum media. Conditioned media was collected and concentrated using Vivaspin columns (10 kDa M_r_ cut off, GE Healthcare, Marlborough, MA) and transfected cells were washed (PBS), detached (EDTA), and centrifuged (1,500 x g, 5 min).

### Immunoblot analysis

Transfected cell pellets were re-suspended (50 μl, 10 min) in detergent lysis buffer (1% IGEPAL, 50 mM Tris-HCL, 150 mM NaCl, pH 7.8) containing HALT protease inhibitor (Pierce/Thermo Scientific, Rockford, IL) then centrifuged (10,000 x g, 10 min) to pellet cell nuclei. Post-nuclear lysate was removed and soluble protein concentration was determined using the Non-interfering assay (G-Bioscience, St. Louis, MO). Soluble proteins (10 μg) and molecular weight markers (10–250 kDa, Li-Cor, Lincoln, NE) were separated on SDS-PAGE gels (10% mini gels, Invitrogen/Life Technologies) then transferred onto nitrocellulose, incubated with FLAG primary antibody (anti-D 1:1000 dilution, GeneCopoeia) followed by goat-anti-mouse IRDye 680LT secondary antibody (1:10,000 dilution, Li-Cor). Protein bands were visualized using an Odyssey Infrared Imaging System (Li-Cor) running Image Studio (Ver 4.0) software. Blots were stripped (NewBlot nitro stripping buffer, Li-Cor) and re-probed with β-actin antibody (1:1000 dilution, Cell Signaling, Danvers, MA) to control for sample-loading and quantification of protein band intensity.

### *In situ* hybridization (ISH)

For ISH, one male mouse (strain C57BL/6J, stock no. 000664, Jackson Laboratory, Bar Harbor, MA) was humanely killed by CO_2_ asphyxiation followed by cervical dislocation at postnatal day 22 (P22). Eyes were removed and fixed (24 hr, 20°C) in 10% neutral buffered formalin (Fisher Scientific) and processed using standard formalin-fixed-paraffin-embedded (FFPE) techniques. ISH was performed using the RNAscope 2.0 FFPE Reagent Kit—RED with custom-synthesized oligonucleotide probes (target probe region 501–1520 bp) designed to the mouse EFEMP1 transcript (NM_146015.2; 2036 bp mRNA) according to the manufacturers instructions (Advanced Cell Diagnostics, Inc. Hayward, CA). Briefly, FFPE microtome (5 μm) sections (RM2255, Leica Microsystems, Buffalo Grove, IL) on glass slides (SuperFrost Plus) were baked (1 hr, 60°C), de-waxed in xylene, dehydrated in ethanol, boiled in citrate buffer, then protease treated (10 μg/ml) in a HybEZ Oven (40°C, 30 min). Pre-treated sections were hybridized with target probes (2 hr, 40°C), followed by signal amplification oligonucleotides (15–30 min, 40°C), then alkaline phosphatase (AP)-conjugated Fast-Red label probe (15–30 min, 20°C). Labeled sections were treated with chromogenic Fast-Red substrate (10 min, 20°C), counterstained (Gill’s Hematoxylin-1/0.01% ammonia-H_2_O), mounted (Clear-Mount), and imaged under a bright-field microscope (BX61, Olympus, Center Valley, PA) fitted with a digital camera (SC-30, Olympus).

## Results

### Glaucoma family

We investigated a three-generation African-American pedigree segregating adult-onset (≥35 years), primary open-angle glaucoma with manifest autosomal dominant transmission in the first two generations (Fig 1). Glaucoma diagnosis was supported by significantly elevated intraocular pressure (IOP) >30 mm Hg with consistent visual field and/or optic nerve abnormalities. Two of the affected individuals (II:7, II:10) were also diagnosed with Bullous keratopathy and age-related cataract (nuclear sclerosis) in the absence of other ocular and/or systemic abnormalities. The glaucoma status of individuals in the third generation is unknown as these relatives fall within the pre-symptomatic age-range (<35 years) and/or did not respond to requests for follow-up examinations. Consequently, the pedigree had an insufficient number of meiotic events with known disease-status to support independent, genome-wide linkage analysis. Instead, an affected father-son-unaffected-daughter trio (I:1, II:4, II:9) was selected for whole exome sequencing.

### Exome variants and exclusion of candidate genes

For all three exome samples, over 98% of total paired-end reads were mapped to the reference genome (S1 Table). Over 86% of mapped reads were present in the captured exomes and the average mean-mapped read depth was >88X with no unexpected gaps in coverage. Over 88% of each exome achieved a read-depth of ≥10X coverage, yielding >46,000 single nucleotide polymorphisms (SNPs), of which >9,000 were non-synonymous and >2,700 were novel.

A review of the exome variants obtained from the trio (Fig 1) using SNP and variation filtering software identified 13 novel, heterozygous, non-synonymous, coding variants that were present in the affected father (I:1) and son (II:4) but not in the unaffected daughter (II:9), consistent with disease causing potential (S2 Table). Interestingly, two of the novel missense variants, one located in *EFEMP1* and one in *CCDC71*, mapped within known loci for monogenic forms of POAG—GLC1H and GLC1L, respectively,—that do not have causative genes identified. No novel variants that co-segregated with disease in the family trio were detected at any of the 19 other known loci for Mendelian forms of POAG (GLC1A-G, GLC1I-K, GLC1M-Q, GLC3A-D) including the eight known causative genes namely, *MYOC* (GLC1A), *CYP1B1* (GLC3A), *WDR36* (GLC1G), *ASB10* (GLC1F), *OPTN* (GLC1E), *NTF4* (GLC1O), *TBK1* (GLC1P), and *LTBP2* (GLC3C). However, known reference-sequence (rs) variants were detected in two genes, *WDR36* (rs144543625) and *OPTN* (rs11258194), that co-segregated with disease in the trio (S3 Table). The latter was previously reported as a risk variant for sporadic cases of POAG [12]. Based on minor allele frequency (MAF) in African-Americans (http://evs.gs.washington.edu/EVS/), rs11258194 in *OPTN* was likely excluded (MAF = 11.75%) and Sanger sequencing confirmed that rs11258194 did not co-segregate with disease in an affected individual (II:7) from the second generation of the pedigree (S3 Table). By contrast, rs1444543625 in *WDR36* was a rare variant found in African-Americans (MAF = 0.0454%) and had not previously been associated with POAG. However, Sanger sequencing again revealed that rs1444543625 did not co-segregate with disease in two affected individuals (II:5, II:10) from the pedigree excluding this variant as disease causing (S3 Table). Similarly, we sought to validate and test the novel variants (S2 Table) for disease co-segregation by performing Sanger sequencing in the first and second generations of the pedigree. This revealed that 11 of the 13 novel variants, including that in *CCDC71* (GLC1L), did not co-segregate with disease across the second generation of the pedigree effectively excluding them as causative mutations (S2 Table). The two remaining, co-segregating variants were each located in *EFEMP1* and *CD248* (tumor endothelial marker 1, endosialin). By contrast with the *EFEMP1* variant, the *CD248* variant (p.Gln402His) was predicted by the SIFT and PolyPhen-2 algorithms to be tolerated or benign with respect to protein function (S2 Table). Furthermore, searches of public tissue-expression and disease databases including BioGPS (http://biogps.org), Ocular Tissue Database (OTDB, http://genome.uiowa.edu/otdb/), and OMIM (www.omim.org), revealed that, unlike *EFEMP1*, *CD248* exhibits minimal expression in the eye and has not previously been associated with ocular disease. While we cannot completely exclude the *CD248* variant, these *in silico* findings indicate that *CD248* is an unlikely candidate gene for POAG in this family. Therefore we focused our further studies on the *EFEMP1* variant.

### *EFEMP1* variant analysis

**Fig 2:**
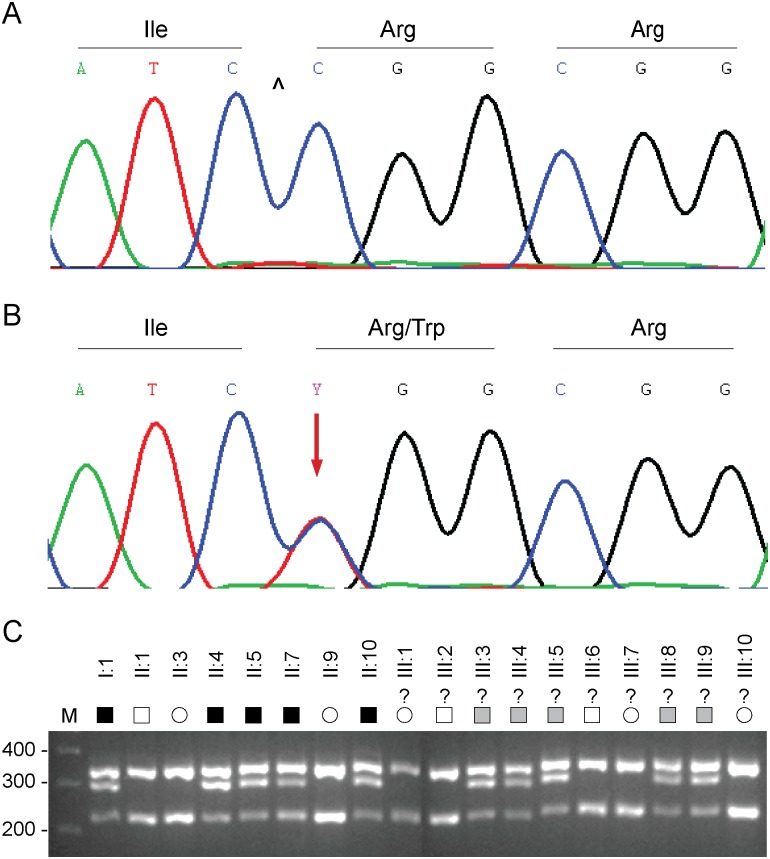
*EFEMP1* variant analysis. (**A**) Sanger sequence trace of the wild-type allele showing translation of arginine at codon 140 (CGG). ^ indicates cut-site for *Hpa* II. (**B**) Sanger sequence trace of the mutant allele showing the heterozygous C-to-T transition (denoted Y by the International Union of Pure and Applied Chemistry [IUPAC]) that is predicted to result in the missense substitution of arginine to tryptophan (TGG). (**C**) Allele-specific restriction fragment length analysis showing loss of an *Hpa* II restriction-site (5’-C^CGG) that co-segregated with affected individuals heterozygous for the C>T transition (300 bp). M, molecular mass markers (bp). Question marks indicate unconfirmed disease status. Filled symbols indicate affected status. Shaded symbols indicate disease haplotype.

The remaining, co-segregating novel exome variant (S2 Table) comprised a heterozygous C>T transition in exon-5 of the gene coding for epidermal growth factor (EGF) containing fibulin-like extracellular matrix protein 1 (*EFEMP1*), also known as fibulin-3, located on chromosome 2p16 (2:56,144,899). This missense change occurred at nucleotide position 418 from the first base of the translation-start codon in the cDNA sequence for EFEMP1 transcript variants 2 and 3 (c.418C>T), and resulted in the loss of an MspI/HpaII restriction site (Fig 2). Sanger sequencing confirmed the presence of the heterozygous c.418C>T change in both of the index affected males (I:1, II:4) and excluded mutations in other exons or splice-sites of *EFEMP1* including a previously identified missense mutation (c.1033C>T, p.Arg345Trp) linked with inherited retinal drusen [11]. Allele-specific PCR amplification and *Msp*1 restriction-fragment-length genotyping further confirmed that the c.418C>T variant co-segregated with known affected but not unaffected relatives in the first two generations of the pedigree (Fig 2). Similar genotyping of the third generation confirmed the presence of the c.418C>T variant in several individuals of unknown disease status with a known affected parent (Fig 1). We note that this variant was not present in one individual in the third generation (III:1) or in her unaffected mother in the second generation (II:3). These observations suggested that the heterozygous c.418C>T variant in *EFEMP1* may be predictive for inheriting POAG in this family.

### Linkage and haplotype analyses

*EFEMP1* is located within the GLC1H locus on chromosome 2p that was identified by linkage analysis of an Afro-Caribbean (Jamaican) family and six Caucasian families [13]. In an effort to confirm linkage to *GLC1H* we genotyped several microsatellite markers on 2p. Lod score (Z) analysis of the abridged pedigree with known disease status (first and second generations only) provided suggestive evidence of linkage at three markers (D2S378, D2S2165, and D2S2113) and at the c.418C>T variant in *EFEMP1* (Z_max_ = 1.81, θ_max_ = 0). While this Lod score falls shy of that desired (Z ≥ 2.0) for linkage to a known disease locus, it is close to the maximum that can be attained by the four affected and three unaffected individuals (7 meioses) in the second generation of the pedigree. Similar analysis of the entire pedigree, including those with inferred disease status in generation three, detected stronger evidence of linkage at marker D2S378 (Z_max_ = 4.21, θ_max_ = 0) and at the c.418C>T variant in *EFEMP1* (Z_max_ = 4.51, θ_max_ = 0) (S5 Table). Further, we sought to define a disease haplotype by identifying recombinant individuals flanking *EFEMP1*. Haplotyping in all three generations of the pedigree detected recombinant individuals III:3 and III:5 at markers D2S2133 and D2S391, respectively, suggesting that the disease lay in the physical interval D2S391-(~24 Mb)-D2S2133 (S1 Fig). This interval completely overlapped that of GLC1H [D2S123-(~10.9 Mb)-D2S2165] and two similar disease intervals [D2S123-(~13.3 Mb)-D2S2397 and D2S391-(~19.2 Mb)-D2S2231] defined by Chinese families segregating autosomal dominant POAG [14,15]. In addition, our interval flanked several common variants associated with complex POAG in populations of different ancestries [16–19]. Taken overall, our variant, linkage and haplotype analyses suggested that *EFEMP1* was a plausible candidate gene for POAG in this family.

### Transient expression studies

**Fig 3:**
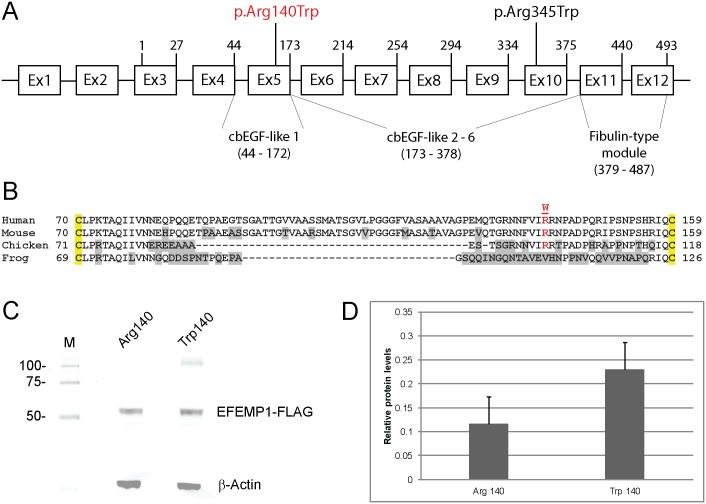
EFEMP1 gene structure and transient expression. (**A**) Schematic of exon organization and protein domains. The gene comprises two non-coding exons (1 & 2) and ten coding exons (3–12) generating at least two transcript variants (2 & 3). Exon 1 and exon 2 are present in transcript variant 2. Exon 2 is skipped in transcript variant 3. Both transcript variants 2 and 3 encode the same 493-amino-acid-protein with 6 calcium-binding (cb) EGF-like domains. The first N-terminal cbEGF-like domain is modified compared with the other five cbEGF-like domains by the insertion of an 88 amino-acid linker region (B). The exon locations of the p.Arg140Trp (p.R140W) and p.Arg345Trp (p.R345W) variants are indicated. (**B**) Amino-acid alignment of the N-terminal cbEGF-like 1 domain (single-letter code) showing cross-species conservation of arginine 140 (R140) located within the 88 amino-acid linker region between conserved cysteine (C) residues (yellow highlight). (**C**) Immunoblot analysis of transfected HEK293T cell-lysates showing expression of wild-type (Arg140) versus mutant (Trp140) EFEMP1-FLAG fusion products. Blots were stripped and re-probed with β-actin to control for sample loading. (**D**) Relative levels of wild-type and mutant EFEMP1-FLAG in transfected cell-lysates normalized to those of β-actin.

The reference sequence for *EFEMP1* (Gene ID: 2202) comprises 12 exons that generate two transcript variants (2 and 3) differing in their upstream untranslated regions (5’-UTRs) but encoding the same 493-amino-acid-protein (www.ncbi.nlm.nih.gov/gene), (Fig 3). The c.418C>T transition occurred at the first base position of codon 140 (CGG>TGG) and was predicted to result in the substitution of tryptophan for a phylogenetically conserved arginine residue (p.Arg140Trp, p.R140W) located in the first of six calcium-binding (cb) EGF-like domains (Fig 3). This represented a non-conservative substitution with the polar/basic side-chain of argenine (-3(CH_2_)-NH-(NH_2_)C = NH) replaced by the non-polar/hydrophobic side-chain of tryptophan (-CH_2_-C = CH-NH-Ph) and was predicted using the SIFT and PolyPhen-2 algorithms to have damaging effects on protein function (S2 Table). In order to gain insights into the functional effects of the p.Arg140Trp amino-acid substitution we undertook transient expression of wild-type (Arg140) and mutant (Trp140) forms of FLAG-tagged EFEMP1 in HEK293T cells followed by immunoblot analysis of cell lysate and conditioned media. FLAG-antibody failed to detect significant levels of mutant or wild-type EFEMP1 in conditioned media suggesting that the expression levels achieved and/or the media concentration method used were insufficient to detect protein secretion. However, we reproducibly detected (n = 4) increased levels (~2-fold) of mutant EFEMP1-Trp140 in transfected cell-lysates compared with those of wild-type (Fig 3) suggesting that Trp140 mutant accumulated abnormally and/or was secreted less efficiently than the wild-type Arg140 protein.

### Ocular localization of EFEMP1 transcripts

**Fig 4:**
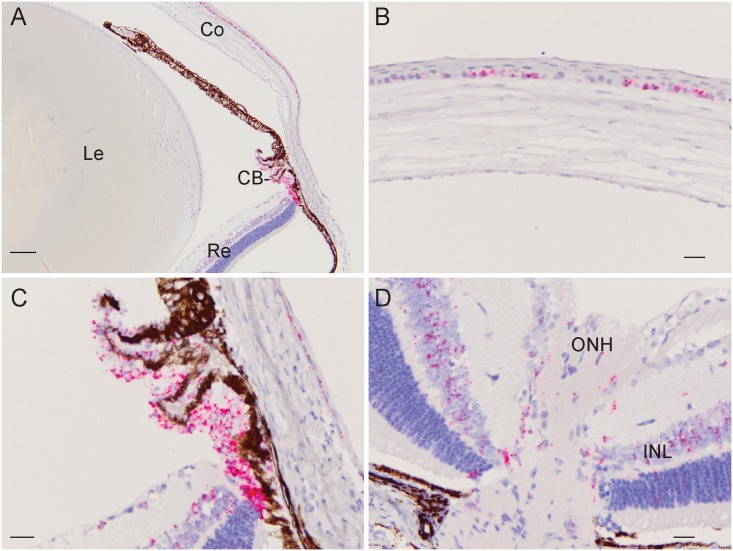
*In situ* hybridization of EFEMP1 transcripts in the mouse eye (P22). (**A**) Low magnification (10x) image (sagittal plane) showing the strongest localization of transcripts (red punctate staining) to the ciliary body (CB) and cornea (Co). (Le—lens, Re—retina). (**B**) Central cornea region (40x) showing localization to the basal epithelium. (**C**) Cilary-body region (40x) showing intense localization to the non-pigmented ciliary epithelium. (**D**) Optic nerve-head (ONH) region of the retina (40x) showing strong localization to the inner nuclear layer (INL). Scale bars: 100 μm (A), 20 μm (B-D).

To determine the ocular expression profile of EFEMP1 mRNA transcripts we conducted ISH analysis of the young mouse eye at postnatal day 22 (Fig 4). Transcripts were most strongly expressed in the ciliary body (non-pigmented epithelium) and cornea (basal epithelium). Lower transcript levels were detected in the inner nuclear layer of the retina and optic nerve-head region with barely traceable levels in the lens. This ocular expression profile was consistent with the EFEMP1 transcript levels detected by microarray analysis of mouse and human eye tissues (BioGPS, http://biogps.org); OTDB, http://genome.uiowa.edu/otdb/).

## Discussion

Previous ocular genetic studies have identified a recurrent, missense mutation in exon-10 of *EFEMP1* (c.1033C>T, p.R345W) associated with Doyne honeycomb retinal dystrophy (DHRD) and/or Malattia Leventinese (MLVT, MIM: 126600) in European and Asian families [11,20–24]. Recently, a novel intronic variant of unknown significance in *EFEMP1* was reported in a DHRD patient [25]. DHRD/MLVT is an autosomal dominant retinal disease characterized by radial deposits of basal-laminar drusen [26]. By contrast, in this study we have identified a novel missense variant in exon-5 of *EFEMP1* (c.418C>T, p.Arg140Trp) co-segregating with an autosomal dominant form of high-tension POAG in an African-American family. *EFEMP1* maps within a known locus (GLC1H) on 2p for monogenic POAG (S1 Fig) in Caucasian, Afro-Caribbean (Jamaican), and Chinese families [13–15]. *EFEMP1* also maps close to common variants associated with complex POAG in Afro-Caribbean (Barbados), African-American, Chinese and South-Indian populations [16–19]. Recently, an intronic SNP (rs1346786) in *EFEMP1* has been associated with optic nerve-head (disc) morphology (central cup area) in Europeans and Asians [27]. Collectively, these genetic findings raise the possibility that variations in *EFEMP1* exhibit pleiotropic effects resulting in variable ocular diseases that may be further influenced by ethnic background.

EFEMP1 (fibulin-3) is a member of the fibulin family of secreted extracellular-matrix/basement-membrane glycoproteins that are characterized by N-terminal tandem arrays of calcium-binding EGF-like domains (cbEGF) followed by a C-terminal fibulin-type module shared with the fibrillins [28,29]. The p.Arg345Trp mutation underlying DHRH/MLVT is located in the cbEGF-like 6 domain adjacent to one of five highly conserved cysteine residues [30]. By contrast, the p.Arg140Trp mutation identified here is located in the first cbEGF-like domain within a protease-sensitive linker region of 88 amino acids (Fig 3) separating the fourth and fifth conserved cysteine residues [31]. Transient expression studies in cultured cells have revealed that the Trp345 mutant is poorly secreted and accumulates in the endoplasmic reticulum (ER) as a result of protein misfolding due to impaired disulfide-bonding resulting in activation of the unfolded protein response [30,32]. Similarly, we found that the Trp140-mutant protein exhibited intracellular accumulation compared with the wild-type (Fig 3), though we are unable to confirm that this was related to impaired secretion. We note however, that *in vitro* introduction of a p.Arg185Trp mutation into the cbEGF-like 2 domain had a much less severe effect on EFEMP1 secretion than that of the p.Arg345Trp mutation [33]. These observations suggest that mutations in different EFEMP1 domains may be tolerated to varying degrees *in vivo* raising the possibility of variable disease presentation, severity and course.

Several experimental approaches have been used to connect EFEMP1 dysfunction with ocular disease. Mice harboring the p.Arg345Trp mutation in *Efemp1* developed progressively larger retinal deposits (between Bruch’s membrane and the retinal pigment epithelium) recapitulating symptoms of human DHRD/MLVT [22,34]. However, mice lacking EFEMP1 did not develop an obvious retinal pathology [35] suggesting that deleterious gain-of-function mechanisms, rather than loss-of-function effects, trigger retinal disease. Gene expression profiling has shown that *EFEMP1* was up-regulated in human trabecular meshwork cells following treatment with transforming growth factor (TGF)-β2—a biomarker for POAG found to be elevated in the aqueous humor of patients [36]. Similarly, *Efemp1* expression was found to be up-regulated in the mouse retina following optic nerve crush—a model system for POAG pathogenesis [37]. Further, *in silico* pathway prediction analysis of positional candidate genes at the GLC1H locus have implicated EFEMP1 in a network of protein-protein-interactions involving other genes associated with POAG [15]. Finally, the pan-ocular expression profile of EFEMP1 in the cornea, ciliary-body, retina, and optic nerve-head, raises the likelihood of its involvement in different ocular pathologies. Possible molecular mechanisms underlying EFEMP1 dysfunction in POAG-relevant tissues include impaired structure and/or function of basement membranes and/or chronic activation of ER-stress leading to cell death.

In conclusion, beyond retinal disease, our data support the notion that *EFEMP1* is a plausible candidate gene for POAG. Further insights regarding the genotype-phenotype complexity associated with *EFEMP1* await future genetic and functional studies.

## Supporting Information

S1 Fig Partial ideogram of chromosome 2. The cytogenetic and physical location of the GLC1H locus is shown in physical relation to *EFEMP1* and other microsatellite (D2S) markers and SNP (rs) markers (boxed) used in linkage or association studies of POAG [13–19].
(TIF)

S1 Table Sample metrics for exome sequencing of the family trio (Fig 1). (DOCX)

S2 Table Summary of novel exome variants found to co-segregate with disease in the family trio (Fig 1). (DOCX)

S3 Table Exome variants found in known genes for Mendelian forms of POAG that co-segregated with disease in the family trio (Fig 1). (DOCX)

S4 Table Gene-specific PCR primers for Sanger sequencing of exome variants in S2 and S3 Tables. (DOCX)

S5 Table Two-point Lod scores (Z). Z values for linkage between the POAG locus in the family (including all three generations) and markers on chromosome 2p listed in physical distance (bp) from the telomere (2p-tel).
(DOCX)
